# Imaging Time Series for the Classification of EMI Discharge Sources

**DOI:** 10.3390/s18093098

**Published:** 2018-09-14

**Authors:** Imene Mitiche, Gordon Morison, Alan Nesbitt, Michael Hughes-Narborough, Brian G. Stewart, Philip Boreham

**Affiliations:** 1 Department of Engineering, Glasgow Caledonian University, 70 Cowcaddens Road, Glasgow G4 0BA, UK; gordon.morison@gcu.ac.uk (G.M.); a.nesbitt@gcu.ac.uk (A.N.); mhnarborough@doble.com (M.H.-N.); 2 Institute of Energy and Environment, University of Strathclyde, 204 George Street, Glasgow G1 1XW, UK; brian.stewart.100@strath.ac.uk; 3 Innovation Centre for Online Systems, 7 Townsend Business Park, Bere Regis BH20 7LA, UK; pboreham@doble.com

**Keywords:** EMI method, EMI discharge sources, classification, Gramian Angular Field, Local Binary Pattern, Local Phase Quantisation

## Abstract

In this work, we aim to classify a wider range of Electromagnetic Interference (EMI) discharge sources collected from new power plant sites across multiple assets. This engenders a more complex and challenging classification task. The study involves an investigation and development of new and improved feature extraction and data dimension reduction algorithms based on image processing techniques. The approach is to exploit the Gramian Angular Field technique to map the measured EMI time signals to an image, from which the significant information is extracted while removing redundancy. The image of each discharge type contains a unique fingerprint. Two feature reduction methods called the Local Binary Pattern (LBP) and the Local Phase Quantisation (LPQ) are then used within the mapped images. This provides feature vectors that can be implemented into a Random Forest (RF) classifier. The performance of a previous and the two new proposed methods, on the new database set, is compared in terms of classification accuracy, precision, recall, and F-measure. Results show that the new methods have a higher performance than the previous one, where LBP features achieve the best outcome.

## 1. Introduction

Condition monitoring of High-Voltage (HV) equipment in power generating plants is essential as any defect puts at risk staff safety as well as the power plant’s operation. Electromagnetic Interference (EMI) is generated due to the presence of electrical or mechanical faults in various equipment types, such as motors, transformers, generators, and switchgear. Conducted and radiated EMI are exploited by EMI experts to gain information on faults type. Consequently, electrical insulation degradation can be identified through EMI diagnosis [1,2]. Popular insulation faults include Partial Discharges (PDs), corona, arcing, sparking, etc. Other non-harmful phenomena, such as exciter, process, and random noise, may also be collected during EMI sensing [3]. The procedure to identify EMI faults by experts is time-consuming and not practical for continuous monitoring. The main goal behind this work is to build an intelligent classification system framework based on EMI expert knowledge. The idea is to train a machine learning model with multiple defect instances measured on HV sites as identified by EMI experts. The trained model is then used to identify the fault or discharge source types within the newly measured signals for the condition monitoring of assets in an HV site.

Previously, authors in [4] developed an initial framework algorithm to classify a limited number of EMI sources. This algorithm demonstrated a high classification performance; however, as the number of signal types increases with a variety of sites, the algorithms performance may degrade. Therefore, this paper attempts to improve this performance by developing new feature extraction techniques whose performance is compared to the previous approach. First, the time series signals are mapped to an image by means of a polar coordinate transformation called the Gramian Angular Field (GAF). This technique was recently introduced by authors in [5] to visualise the time series in the form of an image for improved classification. Next, feature extraction and reduction techniques, called descriptors, are calculated over the GAF image. In this paper, the performance of two descriptors known as the Local Binary Pattern (LBP) and Local Phase Quantisation (LPQ) are evaluated. LBP is an effective and efficient descriptor in image [6,7] and texture classification [8], and LPQ has been shown to be successful in the same applications [9,10].

### Related Work

Fault detection using the machine learning approach has been addressed in many research works [11]. The work related to this paper lies within the scope of insulation fault detection in HV generating power plants. Condition monitoring of HV equipment by means of Machine Learning classification has been previously addressed in the literature. The most popular topic is detection of PD activity [12,13] and PD types [14,15]. Usually, PD is captured in the form of phase-resolved or time-resolved data of a determined pattern. A pattern that can be classified should be characterised by features. Thus, previous research has proposed various feature extraction techniques that can be grouped into signal processing, image processing, statistical methods, and pulse shape methods. The choice of feature extraction technique to employ depends on the data nature and the addressed problem.

A variety of signal processing techniques have been applied to the phase-resolved PD data. For instance, in [16], the authors extracted the minimum and maximum envelopes of the phase versus magnitude plot of multiple PD types. The latter were classified using a Neural Network (NN) approach. In [17], discrete Fourier transform, wavelet packet transform, and cepstral analysis were applied to extract features from PD time signal types in insulation material. An Artificial NN (ANN) was used for classification. The authors in [18] employed cross wavelet transform for the feature extraction of four artificial PD types and classification using ANN.

The image processing technique gained the attention of many researchers in the field. In [19], wavelet decomposition was applied to phase versus magnitude image to classify between corona, PD, surface and cavity discharge. Fractal image features were extracted from the phase-resolved images in [20,21,22] for PD recognition using NN algorithm types.

Statistical measures such as mean, variance, skewness, kurtosis, cross correlation, etc. were calculated as features on the phase-resolved data of five PD defect types in gas-insulated switchgear [23] and in step-up transformer in [24]. Another statistical measure known as q-quantile was applied in [25] to phase data for multiple PD defects recognition in a transformer.

Little attention was given to pulse shape features in the literature until the early 2000s. This method selects the characteristics related to PD pulse shape such as rise and fall time, area, pulse width, and magnitude [26]. The authors in [27] applied similar features to classify PD pulses with various void sizes. Different measures including pulse duration and bandwidth were calculated in [28] as features to discriminate between PD and noise signals by means of classification.

Feature extraction methods belonging to the discussed groups have also been combined. For example, in [29], the authors employed a set of extracted features including pulse shape, statistic measures on the pulse, wavelet energy, and wavelet coefficients for the classification of four PD defect types in cable insulation. In [30], a combination of signal processing and statistical methods was proposed to extract statistical measures from the wavelet coefficients of corona, PD, surface and internal discharge signals.

There is no doubt that the previous work in the literature is of good quality and has been successful. However, the work is limited to laboratory data measured using sensing methods that differ from EMI measurement techniques [31]. In this paper, the authors introduce the classification problem to a completely different perspective of data acquisition, where the data is measured in real-world operating HV equipment using the EMI technique. Furthermore, the signal types addressed in this paper are different from the signal types related to insulation defects that were analysed in the literature.

The paper is structured as follows. The next section summarises the work from data acquisition to model development. The EMI measurement technique is described in Section 3. Section 4 describes the algorithms involved in Machine Learning from feature extraction and reduction to classification. The application of these algorithms to EMI signals is detailed in Section 5. Results and discussion are presented in Section 6, and conclusions are provided in the last section.

## 2. The Proposed Solution

**Figure 1:**
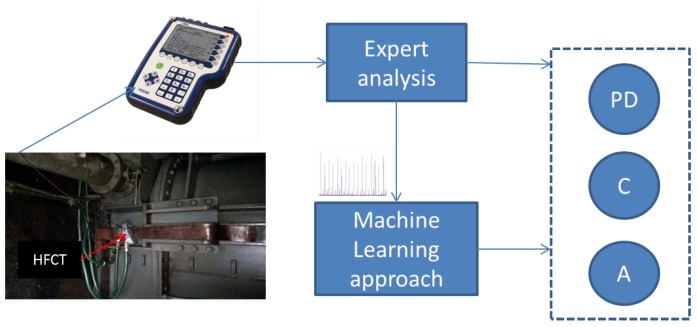
Electromagnetic Interference (EMI) data acquisition using High-Frequency Current Transformer (HFCT) and discharge type identification (e.g., Partial Discharge (PD), Corona (C), and Arcing (A) by EMI experts and their Machine Learning classifications.

Figure 1 outlines the main aspects of this work and the link between them. The idea in this paper is to exploit a database of EMI signals, where each signal contains a discharge type among a variety of discharges, which were identified and labelled by EMI experts. These experts demonstrated knowledge and past experience through forensic investigation and confirmation on previous faulty assets. Thus, it is important to note that this work is based on the assumption that these labels are the ground truth. EMI data measurement and expert analysis will be described further in the next section. As can be seen from Figure 1, the signals are sensed by means of a High-Frequency Current Transformer (HFCT) and are recorded in a device called the PD Surveyor (PDS) 200. The labelled signals are used to develop an intelligent system, based on Machine Learning, that will be able to identify the discharge type as follows. First, feature extraction techniques are employed on EMI signals in order to retrieve features which represent a fingerprint of each discharge type while reducing data dimension. The features for each discharge type along with their relative label are used to train a classification model. Some of the features are not used in the training phase and are preserved to test on the trained model, which will predict the discharge label. The predicted discharge type could be trivial, for example, noise, exciter or important, including severe PD, arcing, corona, etc. This allows further actions, such as trending of an asset’s health, to be taken.

## 3. EMI Measurement Technique

EMI measurement and diagnosis can be used to detect Radio Frequency (RF) energy propagation due to insulation defects in various electrical assets including cables, motors, and generators [1]. The propagated energy can be sensed and measured, in the form of what is referred to as EMI signals, over a wide range of frequencies, from 10 kHz to above 100 MHz. The EMI energy is both conducted and radiated, so the conducted signal is measured using an HFCT usually connected around a neutral earth conduit or on a safety ground connection. EMI methods conforms with the Comitee International Special des Perturbations Radio (CISPR)-16-1-1 standard [32] for results compatibility between EMI measurement instruments. The emitted EMI signals could also result from mechanical defects, such as lost or broken stator and rotor bars, shaft eccentricity, and bearing wear. The EMI technique has the ability to measure the severity, degradation level and location of faults long before identification using more traditional methods. This is beneficial to applications that require system diagnostics. It can differentiate between multiple discharge sources, and supervise activity within adjacent auxiliary equipment as well as within asset parts. Various defects in cables, transformers, Isolated Phase Bus (IPB), generators, stator windings, and exciters have been detected using EMI methods [1]. A Quasi Peak Detector is employed by EMI to measure the radiated signal energy, and to provide a frequency spectrum, referred to as an EMI signature, which is unique to each type of fault and its location [33]. This spectrum is a significant tool for EMI experts in the detection process of the discharge sources along with hearing the complementary audio envelope of the measured signal at a selected frequency. The EMI spectrum in this paper is recorded and displayed by the PDS200device, which acts as a radio receiver that detects the propagated RF energy across a suitable frequency bandwidth of [0–100 MHz]. This instrument also conforms with the CISPR-16-1-1 standard to ensure the filter’s electromagnetic compatibility. The time-resolved signals are also measured by the PDS200 by means of AM demodulation at a frequency of interest, for instance, at the maximum envelope energy. The PDS200 is manufactured by Doble Engineering in Dorchester, UK and Trondheim, Norway.

## 4. Machine Learning Algorithms

This section describes the concept of GAF, LBP, and LPQ algorithms utilised as feature extraction techniques and RF classification algorithms, that are implemented in the proposed approach. Two variations of the GAF algorithm are studied in this work and are described in the following section.

### 4.1. Gramian Angular Field (GAF)

This section introduces two types of GAF algorithm, known as the Gramian Angular Summation Field (GASF) and the Gramian Angular Difference Field (GADF), which are techniques that encode the time series signals into an image. The concept here is to transfer the time series to a polar coordinate space. The Gramian matrix is then formed where each element is calculated by the cosine of the summed angles for GASF or the sine of the subtracted angles for the GADF. This is explained mathematically as follows. First, let vectors be denoted by bold lower case, scalars by lower case and matrices by bold upper case. For *n* real valued observations in a time series $x=x_{1},x_{2},\ldots ,x_{n}$, the latter are first normalised between −1 and 1 as

(1) $$\bar{x}=\frac{x_{i}-max\left(x\right)+\left(x_{i}-min\left(x\right)\right)}{max(x)-min(x)}.$$

This provides angular values in the range [0,π], which will aid in obtaining information granularity in the GAF. The next step is to obtain the polar coordinates which are the cosine angle, ϕ, from the normalised amplitude values and the radius, *r*, from the time stamp *t*, as presented in Equation (2).

(2) $$\left\{\begin{array}{cc} \phi =\cos^{-1}\left({\bar{x}}_{i}\right), & -1\leq {\bar{x}}_{i}\leq 1,{\bar{x}}_{i}\in \bar{x}\\ r=\frac{t_{i}}{N}, & t_{i}\in N \end{array}\right..$$

**Figure 2:**
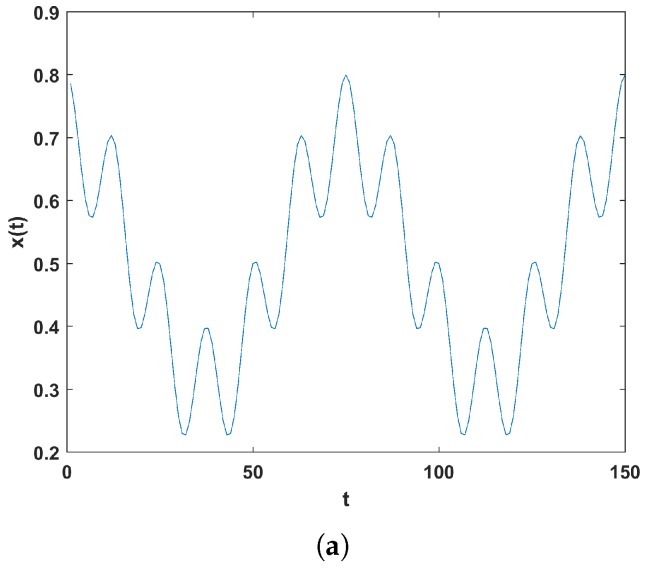

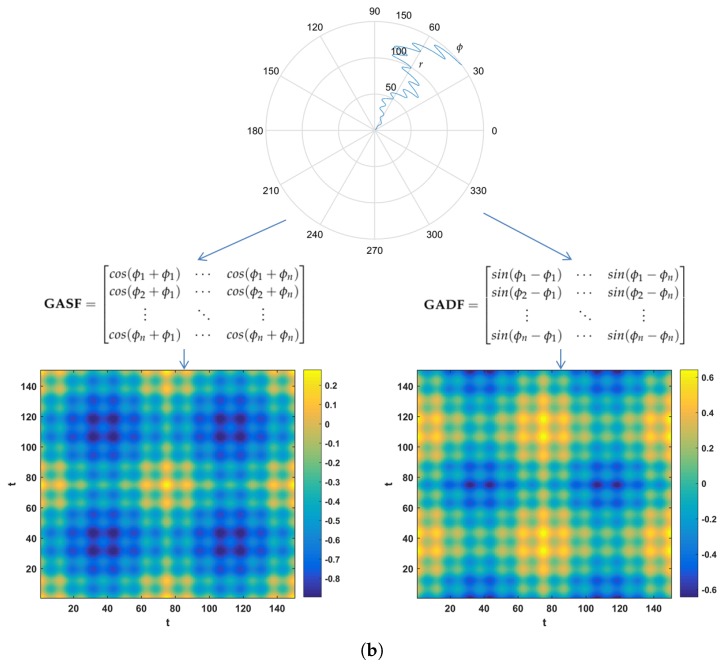
(**a**) Example time series signal; (**b**) polar coordinates mapping of the signal, Gramian Angular Summation Field (GASF), and Gramian Angular Difference Field (GADF) matrix transformation of the signal and their respective image representation.

In Equation (2), *N* is a constant used as a regularisation factor for the polar space span, and is set to N=1 according to [5]. The polar coordinate mapping presents a different perspective of the time series behaviour, in that the time series values bend towards the relative calculated angle as the time increases. This is illustrated in Figure 2b. The polar coordinates representation has two main features: (a) a one-to-one mapping of the time series to the polar coordinate results, so it is bijective, and (b) temporal relations are preserved. The polar coordinates of the normalised time series in the [−1,1] interval fall in the angle boundaries [0,π]. This provides different concentrations of information in the GASF, which should benefit any classification task. Finally, the trigonometric sum can be applied to the inverse cosine (see Equation (3)) between each point, which produces the temporal correlation between different time intervals. To summarise the presented GAF algorithm, Figure 2 illustrates the main transformation of the time signal to an image. First, the polar coordinates are obtained from the time signal using Equation (2). These are then used to calculate the GASF and GADF matrices, with a dimension of i,j=1,…,n, presented in Equations (3) and (4). The obtained matrices can be viewed as images which are plotted at the bottom of Figure 2b.

(3) $$\mathrm{GASF}=\left[\begin{array}{ccc} \cos(\phi_{1}+\phi_{1}) & \cdots & \cos(\phi_{1}+\phi_{n})\\ \cos(\phi_{2}+\phi_{1}) & \cdots & \cos(\phi_{2}+\phi_{n})\\ \vdots & \ddots & \vdots \\ \cos(\phi_{n}+\phi_{1}) & \cdots & \cos(\phi_{n}+\phi_{n}) \end{array}\right].$$

The GADF algorithm is similar to the GASF except that GADF is constructed using the trigonometric difference of the inverse sine as follows:

(4) $$\mathrm{GADF}=\left[\begin{array}{ccc} \sin(\phi_{1}-\phi_{1}) & \cdots & \sin(\phi_{1}-\phi_{n})\\ \sin(\phi_{2}-\phi_{1}) & \cdots & \sin(\phi_{2}-\phi_{n})\\ \vdots & \ddots & \vdots \\ \sin(\phi_{n}-\phi_{1}) & \cdots & \sin(\phi_{n}-\phi_{n}) \end{array}\right].$$

The constructed n×n GASF matrix is exploited as an image for the classifier. However, computation complexity may increase due to the large image size, as it is dependent on the time series length. Therefore, the image is resized and reduced to a convenient standard 224 × 224. This is performed by applying a scale transformation to the original image. Bicubic interpolation is one method of image resizing, where the output pixel value is weighted average calculated over a 4×4 neighbourhood surrounding the input pixel. This method produces a smooth image compared to other interpolation methods and is popular in many image processing algorithms [34].

### 4.2. Local Phase Quantisation (LPQ)

This algorithm is designed for image processing, and it exploits phase information computed from the Fourier phase spectrum of the image. The phase of four low-frequency components are mapped to code words which are embedded in a histogram of features for classification.

Let I(x,y) be an image with m×m dimension. First, the Short-Term Fourier Transform (STFT), with respect to two frequency components *u* and *v*, is performed to retrieve phase information for each pixel of coordinates *x* and *y*, and is calculated on a p×p neighbourhood $N_{x}$ and $N_{y}$, where *p* is the number of pixels, using the following equation:

(5) $$S_{I}\left(u,v\right)=\sum_{y\in N_{y}}\sum_{x\in N_{x}}I\left(x,y\right)\cdot \exp^{-j2\pi (ux+yv)}.$$

In the LPQ calculation, only the phase information, at the first four frequency coefficients ($u_{1}$ to $u_{4}$), is extracted [10], in that $u_{1}=\left(a,0\right)$, $u_{2}=\left(0,a\right)$, $u_{3}=\left(a,a\right)$, and $u_{4}=\left(a,-a\right)$, where a=1/windowsize is a factor of small value that is used in the STFT calculation, where the STFT window size is equal to 7 (see details in [35]). The first four coefficients are formulated in a vector as

(6) $$v=[S_{I}\left(u_{1}\right),S_{I}\left(u_{2}\right),S_{I}\left(u_{3}\right),S_{I}\left(u_{4}\right)].$$

By separating the real and imaginary parts of v, the following is obtained:

(7) w=[Re{v},Im{v}].

Next, the real and imaginary values are quantised using the criteria

(8) $$q_{j}=\left\{\begin{array}{cc} 1 & if\;w_{j}\geq 0\\ 0 & otherwise \end{array}\right..$$

These values are then encoded using binary coding through Equation (9), which provides values in the range of [0–255].

(9) $$LPQ=\sum_{j=1}^{8}q_{j}2^{j-1}.$$

**Figure 3:**
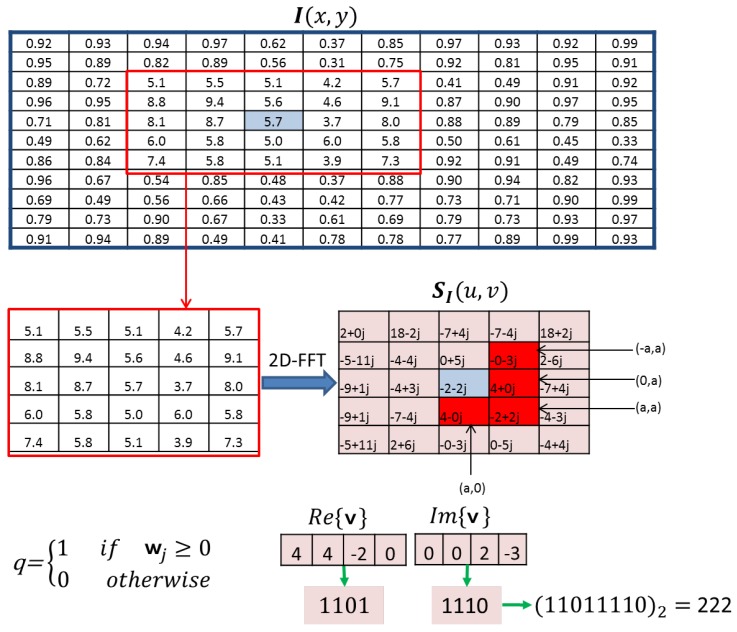
Local Phase Quantisation (LPQ) image encoding of a 2-D image.

Here, the summation is performed on the quantisation of each real and imaginary of the four first frequency coefficients ($u_{1}$ to $u_{4}$), this provides a total of 8 values. The subsequent values of each image pixel are grouped in a histogram, which is normalised then implemented as a 1×256 LPQ feature vector. Figure 3 summarises LPQ calculation steps and shows that the resulting feature vector reduces the data dimension from an image matrix to a vector, while extracting relevant information that could be useful in classification. The 2D-FFT is first calculated on the neighbourhood of the image. The first four frequency components $u_{1}$ to $u_{4}$ are then selected, where the real and imaginary parts of the 2D-FFT are encoded using the criteria in Equation (9). This results in a binary code 11011110, which is converted to a decimal to provide the LPQ value “222”.

### 4.3. Local Binary Pattern (LBP)

**Figure 4:**
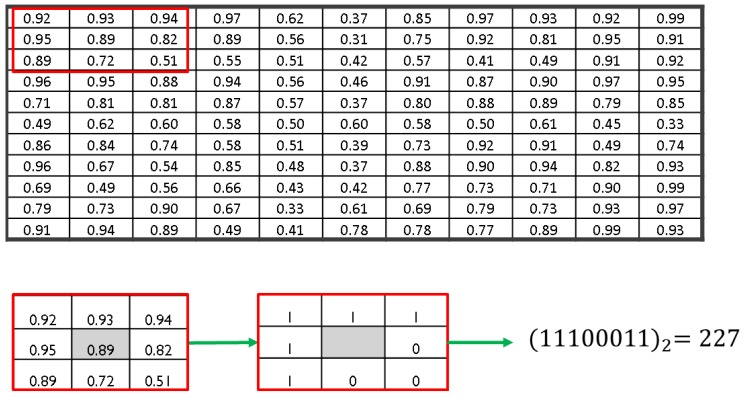
Local Binary Pattern (LBP) image encoding of a 2-D image.

LBP is a binary encoding method for images that extracts non-redundant information and hence reduces the data dimension. The approach is to compare the image pixel values to the neighbouring pixel values resulting in a binary code [36]. Figure 4 shows an example which explains the concept of LBP coding of a 2-D image.

Let “*c*” be the centre pixel that is equally spaced from neighbours “*p*” with a distance “*r*”. The joint difference distribution is calculated as

(10) $$g\approx (g_{0}-g_{c},\ldots ,g_{p-1}-g_{c})$$

where $g_{c}$ and $g_{0}$ to $g_{p-1}$ are the Gray level intensity of the centre pixel *c* and the neighbouring pixels *p*, respectively. The sign of the difference is then used to denote

(11) $$s\left(g_{i}-g_{c}\right)=\left\{\begin{array}{cc} 1 & \mathrm{if}\;g_{i}\geq g_{c}\\ 0 & \mathrm{if}\;g_{i}<g_{c} \end{array}\right.$$

where g can be written in Gray scale format $g\approx (s\left(g_{0}-g_{c}\right),\ldots ,s\left(g_{p-1}-g_{c}\right))$, for the neighbours index i=[0,p]. Finally, an LBP value for each pixel *c*, with the coordinates $\left(x_{c},y_{c}\right);x_{c}\in \left\{0,\ldots ,N-1\right\},y_{c}\in \left\{0,\ldots ,M-1\right\}$ on an n×m image, is calculated as follows:

(12) $$LBP_{p,r}\left(x_{c},y_{c}\right)=\sum_{i=0}^{p-1}s\left(g_{i}-g_{c}\right)2^{i}.$$

This produces a unique value $0\leq c^{\prime }\leq 2^{i}$. LBP values form a histogram with size $p^{2}$. For 8 neighbours, a vector of 256 descriptive features is obtained. It was suggested in [37] that one use only the possible uniform values in the histogram and calculate LBP with 2 points distance. This reduces the feature vector length from 256 to 59 and facilitates computation. If the LBP binary code consists of two 01 or 10 transitions at max, then it is considered uniform. The example LBP provided in Figure 4 is uniform. However, a non-uniform LBP could be noisy and not useful for classification. In contrast, the uniform pattern details the edges, corners, and uniform parts in the image [38]. This could be beneficial in providing significant information on the GAF image of the discharge sources and the differences between them. Further explanation of the uniform LBP is presented in [39]. Because of the mentioned advantages and suggestions, a uniform LBP with the parameters r=2 and p=8 neighbours was implemented in this study.

### 4.4. Random Forest (RF)

The RF classification model is an ensemble of decision trees, trained on random feature sets extracted from labelled data. The randomness property leads to de-correlated trees. The model is created by a combination of components such as weak learners and leaf predictor type. The main drive for using RF in this work is its ability to classify more than two data classes. Furthermore, the low model variances and its parallelism structure makes the RF technique is efficient and overcomes the issue of over-fitting.

The steps for training an RF classifier are as follows.

1. At an initial node, randomly choose *p* feature instances from the overall classifier input *q*, such that *p* is much smaller than *q*.
2. Compute the best split point using Information Gain as
  (13) $$I=H\left(s\right)-\sum_{i\in \{1,2\}}\left|\frac{s^{i}}{S}\right|H\left(s^{i}\right)$$
  given the Shannon Entropy H(s) [40] of the node s, and the child node $s^{i}$.
3. Based on the best split point, split the main node into child nodes and reduce feature instances dimension along the nodes.
4. Repeat Steps 1–3 until a maximum depth l=5 is reached.
5. Repeat Steps 1–4 for k=500 trees of the model. It was found that a larger number of trees yield a higher performance [41].

**Figure 5:**
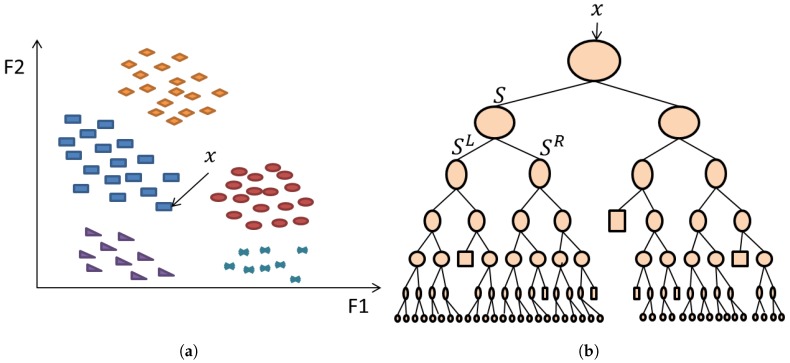
(**a**) Feature space clustering of data instances from different classes (shapes and colours) and (**b**) architecture example of one decision tree classifier.

Figure 5 represents a single decision tree training as explained in the previous steps. In the training phase, the data/label input pair instances are used to optimise tizezhe parameters within each node. The resulting trained model is tested on unseen data and predicts its associated labels based on the rules and parameters generated during the RF model creation. Each trained decision tree $h_{k}$ within the model outputs a prediction. The label that obtains the highest number of votes among all trees is chosen as the predicted label of the input testing instance.

## 5. Application to EMI data

EMI signals were measured, with a sampling rate of 24 kHz and in microvolts, on operating sites using the EMI technique. An EMI expert analysis of these signals was followed in order to label the type of discharge source present in each signal. The outcome of this analysis revealed a total of nine discharge types denoted as Arcing (A), Corona (C), Data Modulation (DM), PD, Process Noise (PN), Random Noise (RN), Exciter (E), minor PD (mPD), and micro Sparking (mS). It is important to note that minor and micro define the discharge level and repetition rate. Details on the EMI signals, including the number of files per dishcarge source, the recorded duration, the asset at which the signal was recorded, and the total samples used for training and testing, obtained through signal segmentation, are presented in Table 1.

First, a previously developed feature extraction technique, called ALIF-Entropy, along with a Multi-Class Support Vector Machine (MCSVM) (see [4]), was applied to this new data. In the previous paper, ALIF Entropy was applied to a different dataset. In this paper, new algorithms, as described in Section 3, are utilised to analyse and classify the new data. The developed model is formed as follows.

1. Divide each time series signal into segments of 2000 samples for ease of GAF computation.
2. Map each time series segment to an image using GASF and GADF algorithms.
3. Resize the images to 224×224 for ease of feature extraction computation.
4. Calculate LPQ and LBP histograms from each image to extract the important features and non-redundant information.
5. Implement the feature histograms with associated labels in the RF classifier.

**Figure 6:**
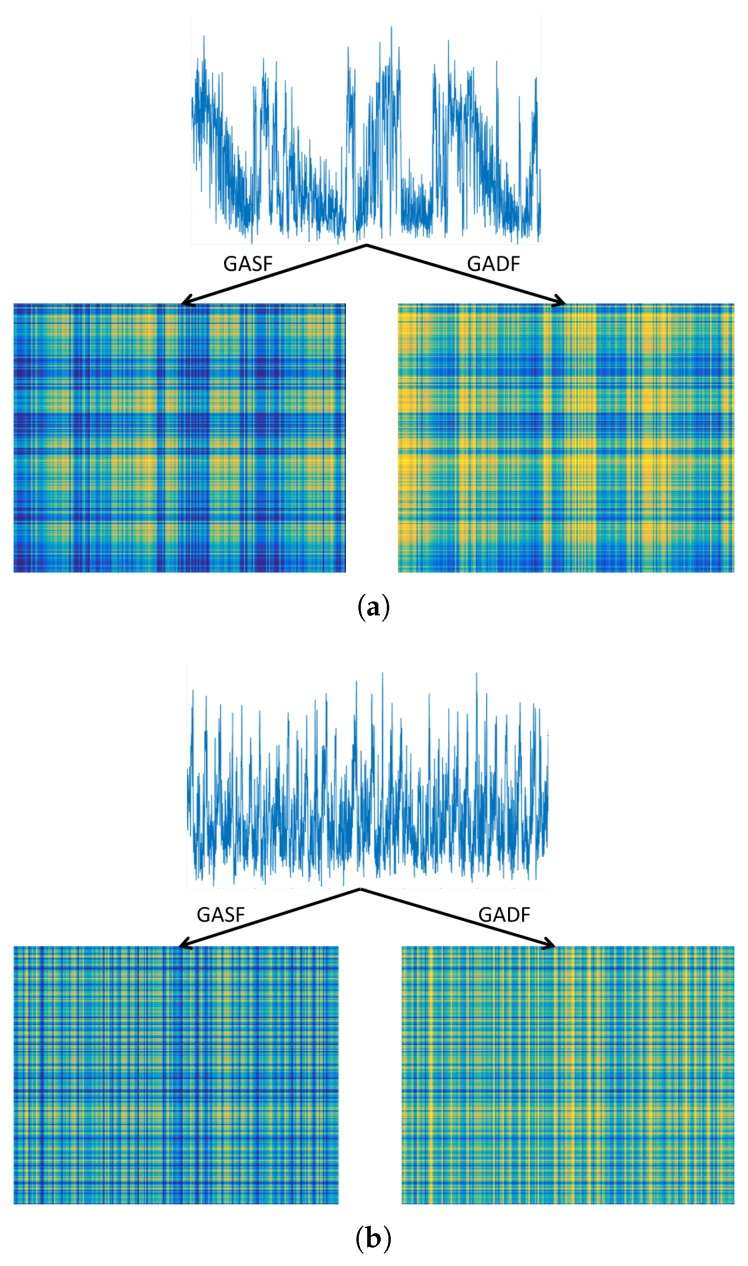
Gramian Angular Summation (GASF) and Gramian Angular Difference (GADF) mapping of (**a**) arcing and (**b**) data modulation.

Figure 6 shows an example time series of A and DM mapped to GADF and GASF images as described in Section 4.1. The feature histogram of LPQ (1×256) and LBP (1×59) was obtained from each image representing a discharge source sample, so a total input feature vector of 1×512 for LPQ and 1×118 for LBP is implemented at a time into the RF classifier. A 10-fold cross validation method was followed in previous papers [42,43] in order to validate the performance consistency of the classifier. This approach is a statistical analysis to assess the performance and skills of machine learning algorithms, and it was proved to be effective for accuracy evaluation [44]. This approach was investigated, and it was found that repeated cross validation did not provide more precise estimates, which is consistent with other cross validation studies [45].

The steps to performing the 10-fold cross validation method are as follows.

**Figure 7:**
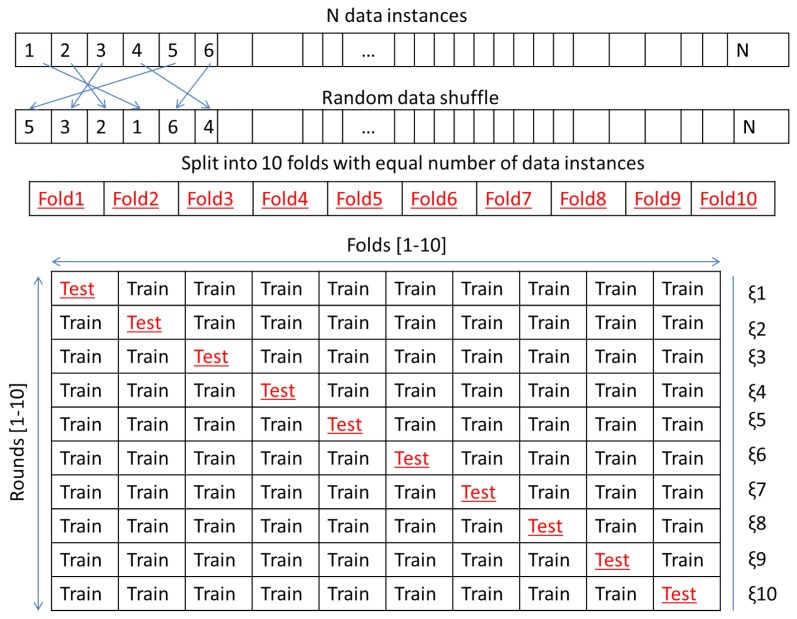
Ten-fold cross validation method for classification of N data inputs (N = 531 in this work).

1. Randomly shuffle the dataset.
2. Split the dataset into 10 groups, in that each group contains samples from each of the 9 classes. For each individual group:
3. Leave the group for testing and use the remaining ones for training.
4. Train and test the model and obtain the classification accuracy.
5. Discard the model, save the accuracy for this fold, and repeat Steps 3–5.
6. Calculate the average accuracy across the saved accuracy ($\xi_{1-10}$ in Figure 7) from each fold.

Here, each data instance is allocated to a group and remains in the group during the 10-fold cross validation procedure, see Figure 7. This indicates that each data instance is provided the occasion to be in the testing set.

The classifier’s performance is evaluated in terms of the most popular evaluation metrics in machine learning, which are classification accuracy (acc) percentage [46,47], precision (pr), recall (rec), and F-measure (*F*) [48,49], which are calculated using Equations (14)–(17), respectively, where tp= true positives, fp= false positives, fn= false negatives, and tpr= total predictions. Classification accuracy is the number of predictions that are correct over all predictions. Precision defines the number of predictions that are actually correct. Recall indicates the number of positives returned by the classifier. The F-measure, also called the F1 Score, represents the balance between precision and recall and is calculated as the harmonic mean of these two measures. A high value of all these measures is preferable and the maximum performance has a value of 1, and 100% for the accuracy. The classification performance is also summarised in a confusion matrix, as followed in previous machine learning-based papers [50,51]. The accuracy for each class is presented in the diagonal of the matrix. Precision class is shown in the bottom row of the matrix and recall in the last column accordingly. The average over all classes for each measure will also be presented in the next section.

(14) $$acc=\frac{tp}{tpr}\cdot 100$$

(15) $$pr=\frac{tp}{tp+fp}$$

(16) $$rec=\frac{tp}{tp+fn}$$

(17) $$F=2\cdot \frac{pr\cdot rec}{pr+rec}$$

(18) $$CI=1.96\times std\left(acc\right)/\sqrt{10}.$$

The evaluations were performed using MATLAB R2017a in a CPU 4 Gb RAM computer.

## 6. Results and Discussion

This section shows and compares the classification findings performed using the proposed feature extraction and classification techniques in this paper and the previously applied technique. The results are presented in Table 2. These results demonstrate that GAF combined with LBP has the ability to extract the fingerprint of each discharge source while achieving a high classification performance and low variance.

**Figure 8:**
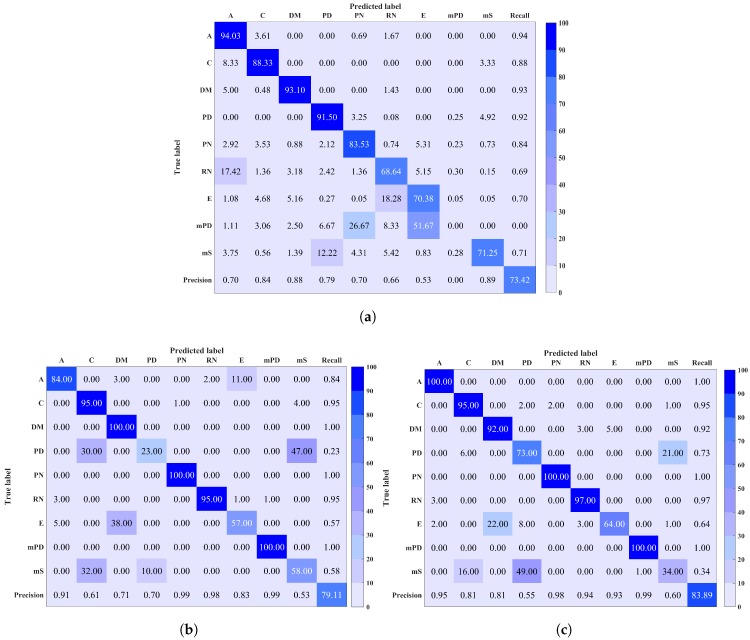
Confusion matrix resulting from (**a**) ALIF-entropy features, (**b**) Gramian Angular Field (GAF) combined with LPQ, and (**c**) GAF combined with LBP. Overall classification accuracy is shown in the bottom right corner.

The confusion matrices of the three approaches are shown in Figure 8. It is observed that the major loss in classification for the ALIF-Entropy based method is in mPD prediction, where mPD signals were mostly classified as RN and E. On the other hand, mPD was successfully predicted at a rate of 100% by both GAF-LPQ and GAF-LBP techniques. However, the confusion matrices highlight the main limitations in PD and mS classifications for LPQ- and LBP-based techniques, respectively. This generates from a confusion between mS, PD, and C. These results are, however, in line with a realistic performance on a large number of discharge sources. Another factor that may impede the classification is noise contamination, as the EMI signals could be overwhelmed by noise causing a change in the signal shape. Therefore, it is of future interest to investigate signal denoising as prior to the feature extraction stage. Another solution is to study other characteristics of the measurements or the signals to distinguish between the discharge sources that are in confusion.

In order to assess the performance of the proposed method with varied window sizes for the signals segmentation, classification results, including the CI of the accuracy across all folds calculated using Equation (18), where 10 is the number of folds, are shown in Table 3. It is observed that employing a window size of 2000 samples provides acceptable results and computation using both LBP and LPQ. Please note that the improvement after 4000 samples is insignificant and not worth considering as the computation is significantly increased.

### Advantages and Limitations of the Proposed Algorithms

The proposed feature extraction algorithms demonstrate the ability to extract relevant fingerprint of the discharge sources and noise signals, which benefits the classification process, and thus provides the potential of automatic EMI diagnosis. The factors that positively influence the classification are as follows. First, setting a number of trees in the random forest to 500 improves the classification accuracy, as it was reported previously that the larger number of trees yield a higher performance. This was confirmed in our analysis where lower classification accuracy was achieved when employing 150 trees only. Second, the analysis of the proposed approach on various window size segments revealed that employing a window of 2000 samples benefits the practicability of this approach with reference to classification performance and computation.

Class imbalance is one of machine learning limitations. This means that the total number of data in some classes is far more than the number of data in others. This issue affects the performance of the proposed GAF-LBP and GAF-LPQ methods as well as the previously proposed ALIF-entropy method, where the support vector machine algorithm does not converge during the training stage. This limitation will be addressed in future work. The authors are currently looking at modifying the cost function in the classification algorithm in order to solve this issue. Furthermore, a weighted version of random forest or support vector machine algorithms will also be considered. Another factor that may affect the discharge signals classification is noise. As the noise level increases in a signal related to a particular fault, the feature extraction and classification could be more challenging. Denoising techniques could be considered for highly noisy signals representing a discharge source. A typical approach would be to measure the noise level in a signal and compare it to a particular threshold. If the noise level falls below the threshold, then the signal should be denoised. To summarise, class imbalance and noise negatively influence the performance of the proposed approach.

## 7. Conclusions

This work introduces a new algorithmic approach to EMI discharge source classification that improves upon previous work for an increased number of discharge source types. The novel approach is based on time series imaging using the GAF method combined with image-based feature extraction techniques known as LBP and LPQ. The algorithm performance was evaluated using the 10-fold cross validation approach and the mean, variance, and CI of the classification accuracy across all folds. Classification results show a successful improvement compared to previous work using both techniques, and LBP-based work achieved the best performance. One limitation in the findings was the confusion of PD and mS signals. Investigation on other aspects of the measured signal, such as frequency or denoising, should be considered in this case. Despite this drawback, the proposed approach could significantly aid in and contribute to an easier and faster method of EMI diagnostics compared to the traditional method. To conclude, the gain in classification performance is a gain in confidence and a motivation to consider the implementation of this approach in an EMI diagnosis instrument. Future work will consist of the full cross validation of the developed algorithms with synthetically generated laboratory captured and real world data sets in the presence of different noise types and levels, as well as other sources of interference, which could lead to a multi-label problem, where multiple EMI sources could be identified in a single signal that carries more than one EMI source. Statistical analysis on how noise affects the algorithm performance will also be employed.

## Figures and Tables

**Table 1:** Detected discharge sources in each site.

Event	no. files	Duration	Asset	Total Training/Testing Samples
Arcing	1	10 s	Boiler Feed Pump	59
Corona	1	10 s	Generator	59
Data Modulation	1	10 s	Boiler Feed Pump	59
PD	1	10 s	Boiler Feed Pump	59
Process Noise	1	10 s	Generator	59
Random Noise	5	1 s	Boiler Feed Pump	59
	1	5 s	Steam Turbine Generator	
Exciter	1	10 s	Generator Step-Up	59
mPD	1	10 s	Generator	59
mS	2	1 s	Salt Water Pump	59
	1	8 s		

**Table 2:** Average classification performance results. Best performance is in bold font.

Feature Extraction Technique	Accuracy %	Variance	Precision	Recall	F-Measure
ALIF-Entropy	73	0.002	0.66	0.73	0.69
GASF-LPQ and GADF-LPQ	79	**0.001**	0.81	0.79	0.80
GASF-LBP and GADF-LBP	**84**	**0.001**	**0.84**	**0.84**	**0.84**

**Table 3:** Average classification accuracy and 95% CI results with varied window size of segments. Best performance is in bold font.

Feature Extraction Technique	1000 Samples	2000 Samples	4000 Samples
GASF-LPQ and GADF-LPQ	**71**% CI{70.95,71.045}	79% CI{78.97.,79.029}	78% CI{77.96,78.037}
GASF-LBP and GADF-LBP	70% CI{69.97,70.027}	**84%** CI{83.97,84.023}	**87%** CI{86.98,87.018}
